# The Emerging Global Tobacco Treatment Workforce: Characteristics of Tobacco Treatment Specialists Trained in Council-Accredited Training Programs from 2017 to 2019

**DOI:** 10.3390/ijerph18052416

**Published:** 2021-03-02

**Authors:** Christine E. Sheffer, Abdulmohsen Al-Zalabani, Andrée Aubrey, Rasha Bader, Claribel Beltrez, Susan Bennett, Ellen Carl, Caroline Cranos, Audrey Darville, Jennifer Greyber, Maher Karam-Hage, Feras Hawari, Tresza Hutcheson, Victoria Hynes, Chris Kotsen, Frank Leone, Jamie McConaha, Heather McCary, Crystal Meade, Cara Messick, Susan K. Morgan, Cindy W. Morris, Thomas Payne, Jessica Retzlaff, Wendy Santis, Etta Short, Therese Shumaker, Michael Steinberg, Ann Wendling

**Affiliations:** 1 Tobacco Treatment Specialist Training Program, Roswell Park Comprehensive Cancer Center, Buffalo, NY 14263, USA; ellen.carl@roswellpark.org; 2 Tobacco Treatment Specialist Training and Certification Program at College of Medicine, Taibah University, Madinah 42353, Saudi Arabia; aalzalabani@gmail.com; 3 Tobacco Treatment Specialist Course, College of Medicine, Florida State University, Tallahassee, FL 32306, USA; andree.aubrey@med.fsu.edu; 4 Tobacco Dependence Treatment Training, King Hussein Cancer Center, Amman 11941, Jordan; rbader@khcc.jo (R.B.); fhawari@khcc.jo (F.H.); 5 Rutgers Tobacco Dependence Program, New Brunswick, NJ 08903, USA; beltrecl@sph.rutgers.edu (C.B.); steinbmb@rwjms.rutgers.edu (M.S.); 6 Tobacco Treatment Specialist Training Program, Mayo Clinic Nicotine Dependence Center, Rochester, MN 55905, USA; bennett.susan@mayo.edu (S.B.); shumaker.therese@mayo.edu (T.S.); 7 Tobacco Treatment Specialist Training Program, Center for Tobacco Treatment Research and Training, University of Massachusetts Medical School, Worcester, MA 01655, USA; caroline.cranos@umassmed.edu; 8 BREATHE Online Tobacco Treatment Specialist Training Program, College of Nursing, University of Kentucky, Lexington, KY 40504, USA; audrey.darville@uky.edu; 9 Duke-UNC Tobacco Treatment Specialist Training Program, Duke Smoking Cessation Program, Duke Cancer Center, Durham, NC 27705, USA; jennifer.greyber@duke.edu; 10 Tobacco Treatment Training Program, The University of Texas MD Anderson Cancer Center, Houston, TX 77030, USA; maherkaram@mdanderson.org; 11 Tobacco Treatment Specialist Training Program, University of Kansas Medical Center, Kansas City, KS 66160, USA; thutcheson@kumc.edu; 12 Tobacco Treatment Education & Training Program, MaineHealth Center for Tobacco Independence, Portland, ME 04101, USA; vhynes@mainehealth.org; 13 Tobacco Treatment Specialist Training Program, Memorial Sloan Kettering Cancer Center, New York, NY 10022, USA; kotsenc@mskcc.org; 14 Comprehensive Smoking Treatment Program, Perelman School of Medicine, University of Pennsylvania, Philadelphia, PA 19104, USA; frank.leone2@pennmedicine.upenn.edu; 15 Tobacco Treatment Specialist Training and Certificate Program, School of Pharmacy, Duquesne University, Pittsburg, PA 15282, USA; mcconahaj@duq.edu; 16 Tobacco Treatment Specialist Certification Program, The Breathing Association, Columbus, OH 43203, USA; heather.mccary@breathingassociation.org; 17 Tobacco Prevention and Control Program, Wellness and Prevention Department, Alaska Native Tribal Health Consortium, Anchorage, AK 99508, USA; cdmeade@anthc.org; 18 National Jewish Health^®^, Denver, CO 80206, USA; messickc@njhealth.org; 19 Tobacco Treatment Training Program, School of Dentistry, West Virginia University, Morgantown, WV 26506, USA; smorgan@hsc.wvu.edu; 20 Rocky Mountain Tobacco Treatment Specialist Training Program, Department of Psychiatry, University of Colorado Anschutz Medical Campus, Aurora, CO 80045, USA; cindy.morris@cuanschutz.edu; 21 ACT Center for Tobacco Treatment, Education and Research, University of Mississippi Medical Center Cancer Institute, Jackson, MS 39213, USA; drtjp123@gmail.com; 22 Council for Tobacco Treatment Training Programs, Inc., Madison, WI 53704, USA; jretzlaff@reesgroupinc.com (J.R.); wsantis@ctttp.org (W.S.); 23 Optum’s Quit for Life Program^®^, Eden Prairie, MN 55344, USA; etta.short@rallyhealth.com; 24 Tobacco Cessation Program, Healthways, A Sharecare Company, Franklin, TN 37067, USA; ann.wendling@sharecare.com

**Keywords:** smoking cessation, health care professional training, tobacco dependence treatment, evidence-based practice

## Abstract

Tobacco use is projected to kill 1 billion people in the 21st century. Tobacco Use Disorder (TUD) is one of the most common substance use disorders in the world. Evidence-based treatment of TUD is effective, but treatment accessibility remains very low. A dearth of specially trained clinicians is a significant barrier to treatment accessibility, even within systems of care that implement brief intervention models. The treatment of TUD is becoming more complex and tailoring treatment to address new and traditional tobacco products is needed. The Council for Tobacco Treatment Training Programs (Council) is the accrediting body for Tobacco Treatment Specialist (TTS) training programs. Between 2016 and 2019, *n* = 7761 trainees completed Council-accredited TTS training programs. Trainees were primarily from North America (92.6%) and the Eastern Mediterranean (6.1%) and were trained via in-person group workshops in medical and academic settings. From 2016 to 2019, the number of Council-accredited training programs increased from 14 to 22 and annual number of trainees increased by 28.5%. Trainees have diverse professional backgrounds and work in diverse settings but were primarily White (69.1%) and female (78.7%) located in North America. Nearly two-thirds intended to implement tobacco treatment services in their setting; two-thirds had been providing tobacco treatment for 1 year or less; and 20% were sent to training by their employers. These findings suggest that the training programs are contributing to the development of a new workforce of TTSs as well as the development of new programmatic tobacco treatment services in diverse settings. Developing strategies to support attendance from demographically and geographically diverse professionals might increase the proportion of trainees from marginalized groups and regions of the world with significant tobacco-related inequities.

## 1. Introduction

While tobacco control efforts have made progress in reducing the prevalence of cigarette smoking, Tobacco Use Disorder (TUD) is one of the most common substance use disorders in the world [1,2] and tobacco use remains a leading cause of preventable death and disease, responsible for more than 8 million deaths worldwide every year [3]. Over 600,000 of these deaths annually are from secondhand smoke, including 30% children [4,5]. Moreover, tobacco use has a disproportionate impact on lower socioeconomic groups [3]. Over 80% of the 1.3 billion tobacco users worldwide live in low or middle-income countries with some of the highest rates of tobacco-related death, disease, and health care costs [6]. If current consumption rates continue, smoking cigarettes alone is projected to kill 1 billion people in the 21st century [7,8]. Tobacco use also imposes substantial global economic burdens. The average cost of smoking cigarettes globally is 1.8% of the gross domestic product (GDP) [9], but this burden is not equally shared. Nearly 40% of this burden is shouldered by developing countries, causing significant financial liability on already-limited budgets [10]. For example, Jordan loses 6% of its GDP to tobacco use annually [11]. Accordingly, tobacco use is one of the leading causes of health inequities in the world [12,13].

The World Health Organization (WHO) Framework Convention on Tobacco Control (FCTC), developed to address the worldwide proliferation of tobacco products, is focused on international efforts to reduce demand for and supply of tobacco products [14]. At present, 182 parties covering 90% of the world have joined the treaty. Article 14 of the FCTC states that each party should take evidence-based measures to promote tobacco cessation and provide treatment for tobacco dependence, but little progress has been made in many parts of the world [15]. For instance, the Eastern Mediterranean has some of the highest tobacco smoking prevalence rates in the world [16,17]. Over 42% of men in the United Arab Emirates [18], and 70% of men in Jordan smoke cigarettes [11]. In Lebanon, 50% of men and women smoke cigarettes [16]. Nearly 20% of young people in the Eastern Mediterranean smoke tobacco in waterpipes [17]. The Gulf Cooperation Council (GCC: United Arab Emirates, Saudi Arabia, Qatar, Kuwait, Oman, Bahrain) has prioritized the reduction of tobacco smoking [19,20]. The WHO Regional Committee for the Eastern Mediterranean lists tobacco control as a crucial part of a regional framework for action to control premature death from non-communicable diseases [3,21].

TUD is a complex behavior influenced by powerful biological, psychological, social, and cultural factors [22,23,24]. Most tobacco users want to quit and many make attempts to quit every year, but few attempts result in long-term abstinence [25,26]. For example, in Jordan, 57% of cigarette smokers intend to quit in the next year and 42% in the next 30 days [27]. In the US, 70% of cigarette smokers want to quit and more than half make a quit attempt each year, but less than 10% of these attempts result in 6 months of abstinence [26]. Nearly 80% of tobacco users have difficulty quitting, experience withdrawal symptoms, and continue to use tobacco despite the knowledge of the harm [23]. Moreover, the complexity of treating TUD is increasing as new tobacco products are introduced and combined with traditional tobacco products worldwide. In addition to traditional cigarettes, cigars, cigarillos, and a wide variety of smokeless tobacco products, there are hundreds of different electronic nicotine delivery systems (ENDS, e.g., e-cigarettes, vaping devices) with thousands of flavors [28], dissolvable tobacco products, and heat-not-burn delivery systems [29]. (See Supplementary Materials for examples of tobacco product use common in certain regions of the world.) Detailed knowledge of this changing landscape is required to engage in meaningful and effective discussions with tobacco users.

Evidence-based treatment of TUD dramatically increases the chances of achieving long-term abstinence [30,31,32], but utilization of evidence-based approaches worldwide remains very low [25]. The highest standard of care for TUD is evidence-based behavioral treatment provided by highly skilled practitioners, guided by treatment manuals, and combined with pharmacotherapy [33,34,35,36,37,38]. In the US, one of the wealthiest countries in the world, less than one third of quit attempts are aided by evidence-based treatment and less than 5% receive the highest standard of care [25,26]. Greater use of evidence-based treatments would result in more individuals achieving long-term abstinence more quickly and reduced tobacco-related death, disease, and economic loss worldwide.

Lack of treatment access is a primary contributor to low utilization of evidence-based treatments for TUD [24,39]. In addition to lack of clinician time [40,41,42], internationally one of the most common barriers to providing treatment is a lack of training in the treatment of TUD [15,40,41,42,43,44,45,46,47,48,49]. Advanced provider training is linked with improved tobacco treatment skills and clearly improves patient outcomes [36,37,50,51]. Moreover, lack of provider training disproportionately affects individuals with complex TUD presentations such as individuals using multiple tobacco products, with mental health conditions, with cancer and other chronic health conditions, and pregnant women, all of whom show clear benefits from specialized TUD treatment [52,53,54].

Systematic, brief intervention models are recommended by many clinical practice guidelines [31,55,56,57] and have significantly increased the number of cigarette smokers identified and advised to quit; nevertheless, after decades of implementation, few tobacco users are provided with evidenced-based assistance [26,58]. Complementary models, however, that incorporate specialist treatment into brief intervention models (e.g., Ask, Advise, Connect) and are tested in real-world settings can dramatically increase the proportion of patients who receive treatment [34,36,59,60,61,62]. Recent advances in lung cancer screening have also provided opportunities for highly trained specialists to reach high-risk populations [63].

Although there are dozens of different tobacco treatment training programs worldwide, little is known about the characteristics of individuals who seek advanced training in the treatment of TUD [15,44,64]. Established in 2008, the Council for Tobacco Treatment Training Programs (www.ctttp.org (accessed on 24 February 2021)) has developed an interdisciplinary approach to implementing training standards [65] for Tobacco Treatment Specialists (TTSs) with the goal of ensuring that TTSs are prepared to meet the needs of dynamic, diverse, and complex populations of tobacco users in diverse settings. As the number of accredited training programs and trainees has grown, little is known about the nature of the accredited programs and the trainees and whether the Council and these training efforts are well-positioned to meet the needs of tobacco users in diverse settings. This study of the data collected by the Council from 2016–2019 aimed to examine the nature of the training programs, the characteristics of the trainees, and to make recommendations for developing a tobacco treatment workforce that meets tobacco treatment needs worldwide.

## 2. Materials and Methods

Council accreditation requires a TTS training program to complete a formal application and review process, often with guidance provided by Council reviewers. Each application describes the nature of the training program, the target treatment population, and other unique characteristics in detail, including its goals and objectives, faculty, and process for inviting professionals, who serve culturally and linguistically diverse populations. The application requires the program to demonstrate how they teach each of the required skills, how they engage in self-evaluation, and how they evaluate trainee learning. The application is reviewed by the Board of Councilors, experts in the field, and accreditation is awarded when the Board of Councilors determines that the training program meets all the training standards. Continued accreditation is subject to annual review.

All Council-accredited training programs meet the same minimum training standards, but differ in their missions, settings, training models, training modalities, and the tailored focus they provide trainees. Successful completion of a Council-accredited TTS training program is necessary to be eligible for the National Certificate of Tobacco Treatment Practice (NCTTP) (https://www.naadac.org/NCTTP (accessed on 24 February 2021)).For instance, some programs, such as Optum’s Quit for Life, National Jewish Health^®^, MercyCare, and Healthways, train professionals only within their organizations. While most programs train professionals on their campuses, many programs also travel to other sites, nationally and internationally, to provide training. From 2016–2019 most programs provided the bulk of the training via in-person workshops.

In 2016 and 2017, the Council, in collaboration with the training programs, implemented mandatory reporting. In 2016, all programs were required to report the number of trainees trained annually. In 2017, the collection of a minimal common set of data from trainees was implemented. This data is collected by the programs, generally prior to training, and reported in a de-identified manner to the Council by January 31 following the reporting year. The minimal data set includes basic demographic information as well as professional background, work setting, experience in the treatment of tobacco dependence, and reasons for seeking training including: “I want to treat tobacco users,” “My organization is requiring me to,” “I want to get certified,” “I want to learn more about the field of tobacco treatment,” and “I want to do research.” Trainees can identify more than one reason.

Data was compiled by the Council management (Jessica Retzlaff) and analyzed by Drs. Carl and Sheffer. Descriptive analyses were conducted for all variables (range, means, standard deviations, frequencies, percentages). Pearson correlation coefficient was calculated between the number of hours per week devoted to tobacco treatment and the number of years working in tobacco treatment. Professional background and work setting were categorized within common professional themes. For example, registered, licensed, surgical, hospice, and palliative care nurses were categorized under nursing.

## 3. Results

Between 2016 and 2019, *n* = 7761 trainees completed Council-accredited TTS training programs; *n* = 2274 in 2019; *n* = 1829 in 2018; *n* = 1888 in 2017, and *n* = 1770 in 2016. Trainee characteristics were available for 86.8% of trainees in years 2017–2019 (*n* = 5203 total; *n* = 2227 in 2019; *n* = 1656 in 2018; and *n* = 1320 in 2017). Trainees resided in six international regions: North America (*n* = 4519), Middle East (*n* = 297), Europe (*n* = 38), Asia (*n* = 15), Africa (*n* = 11), and Australia (*n* = 1); were predominantly White (69.1%), women (78.7%), and had completed at least some college education (93%). See Table 1. From 2016 to 2019, the annual number of trainees increased by 28.5%. See Table 2. A complete list of training programs accredited through 2020 is included in Table 3. 

Trainee professional background and work setting were diverse. (See Table 4 and Table 5) About 80% of respondents were from 13 professional backgrounds and nearly 20% were from 16 less frequently reported professional backgrounds. The response rate for this item was relatively low, 74%, which might reflect difficulty in answering the question among trainees without mainstream professional backgrounds. Over half (50.4%) of trainees worked in larger institutions such as hospitals, medical centers, or academic medical centers; however, 42.7% worked in one of seven other settings, and 6.9% worked in one of 17 less frequently reported settings.

The reasons for attending training, experience of trainees in the field of tobacco treatment, and the amount of time devoted to tobacco treatment indicate that most trainees were new to the tobacco treatment workforce. The most frequently reported reasons for training were to implement tobacco treatment services (61%), to learn (56.6%), and to become certified (52.1%). A meaningful proportion of trainees (15%) attended to improve their tobacco-related research and evaluation skills. Two-thirds (*n* = 2583; 66.2%) were new to the field having worked in tobacco treatment for 1 year or less. Nearly half of those working 1 year or less (44.5%; *n* = 1735) reported 0 years of working in tobacco treatment. Another 19.9% (*n* = 777) of trainees had worked in tobacco treatment for 5 years or less. In terms of time spent delivering tobacco treatment services, 44.7% (*n* = 1874) spent two days or less working in tobacco treatment. The number of hours per week working in tobacco treatment was positively correlated with the number of years working in tobacco treatment (Pearson correlation 0.24, *p* < 0.0001). The more years working in tobacco treatment, the more hours per week trainees reported working in tobacco treatment. See Table 1.

## 4. Discussion

The Council-accredited TTS training programs, diverse in terms of mission, setting, and training modalities, appear to be contributing to the development of an emerging, highly trained tobacco treatment workforce. The number of Council-accredited TTS training programs and trainees has steadily increased in the last decade. Findings show a 28.5% increase in the number of trainees from 2016–2019. This increase coincides with the addition of eight new training programs from 2016–2019, two of which are located in the Eastern Mediterranean, a region with remarkably high tobacco smoking prevalence rates and a tremendous need for accessible, high quality, tobacco treatment. Training is an important step in developing the resources needed to provide access to tobacco treatment [15] and understanding the nature of the training programs and the characteristics of the trainees is key to developing a tobacco treatment workforce that meets tobacco treatment needs worldwide.

Trainees were from diverse professional and educational backgrounds and worked in a wide variety of medical, behavioral health, public health, and community settings which suggests that the interdisciplinary approach to developing the training standards was effective in meeting the needs of diverse professionals. Nonetheless, one of the most striking characteristics of trainees was their inexperience with tobacco treatment. Over 40% were not currently providing any tobacco treatment, and two-thirds had been providing tobacco treatment for 1 year or less. Yet nearly two-thirds of trainees intended to implement tobacco treatment services in their setting, and more than half were seeking TTS certification. This suggests that the Council-accredited training programs are indeed developing an emerging workforce with professionally diverse backgrounds seeking to identify as TTSs as well as contributing to the development of programmatic tobacco treatment services.

The demographic background of this emerging workforce, however, has not been particularly diverse. Trainees were primarily White (70%) and female (80%), about 14% Black or African American, and centered in North America, with little change during the observation period. While the two new programs in the Eastern Mediterranean have the potential to increase diversity, efforts to enhance diversity and inclusion within all programs is needed. A more diverse tobacco treatment workforce is key to meeting the needs of the many marginalized groups that experience tobacco-related disparities [66]. Coordinated efforts to increase trainee demographic and regional diversity are needed and well within the mission of the Council.

The primary catchment areas for many programs has been regional; however, the onset of the COVID-19 pandemic from SARS-CoV-2 virus in early 2020 caused nearly all programs to cancel in-person workshops and develop virtual training opportunities that have the potential to reach more diverse trainees. Virtual training opportunities also might enable distant groups to benefit from the expertise of particular training programs. For instance, health care settings across the world that treat individuals from the Eastern Mediterranean for TUD can now potentially access virtual training tailored to treating this population. Analyses of trainee data in the coming years will provide insight into the impact of the proliferation of virtual TTS training opportunities on the number and characteristics of trainees.

Nonetheless, many programs have a special niche within their institutions or within their state or regional tobacco control programs and thus their missions are not necessarily focused on volume or reach. With a few exceptions, the programs that have been training TTSs for the longest period of time tended to train a higher proportion of trainees. For instance, the University of Massachusetts program trained 27% of the trainees and the Mayo Clinic program trained 11.8% of the trainees between 2016–2019. These programs have dedicated staff, were established about 20 years ago, and travel nationally and internationally, which is likely to contribute to a higher volume of trainees.

Training a workforce in the evidence-based treatment of tobacco dependence is needed to improve access to effective treatment for TUD [14], particularly in low and middle-income countries. To this end, support and resources to develop training programs and fund trainee attendance is needed. Funding from Global Bridges was used to support attendance of some trainees at the King Hussein Comprehensive Cancer Center, but that is likely to be the exception. While the Council does not collect data on how trainees fund attendance, the high proportion of trainees who attend because they are sent by their employers as well as the high proportion of trainees who work in larger institutions such as hospitals, medical centers, and academic medical centers suggests that a large proportion of trainees’ attendance is paid by employers. New virtual training opportunities have the potential to accelerate accessibility, but barriers remain including the cost of tuition, engaging in 30–40 h of training time, and access to high-speed internet. Within the mission of the Council is developing strategies to support attendance from demographically and geographically diverse professionals; doing so might increase the number of trainees from less well-supported organizations, from marginalized groups, and from low and middle-income countries. Asynchronous virtual training opportunities and blended (synchronous and asynchronous) training can help professionals train around busy work schedules. Ensuring professionals are released from duties or compensated for the time they devote to training would also help improve access. In the future, collecting the source of support for attendance might be an important new addition to the Council’s minimum data set.

The strengths of this study include the characterization of multiple years of trainees who completed TTS training within a large group of training partners, accredited by one organization with one set of training standards. The large number of trainees allows for some generalization. Being a descriptive study, however, there are no methods for making causal conclusions about the nature of the programs and the characteristics of the trainees.

## 5. Conclusions

A highly skilled tobacco treatment workforce is a critical component in building the infrastructure and systems of care to increase the accessibility of treatment for TUD. The treatment of TUD is becoming more complex and tailoring treatment to address new and traditional tobacco products is needed. The Council is committed to the development and proliferation of training that meets the needs of a dynamic, diverse, and complex population of tobacco users. Council-accredited TTS training programs are contributing to the development of an emerging workforce prepared for the tobacco treatment challenges of the 21st century. Developing strategies to support attendance from demographically and geographically diverse professionals might increase the number of trainees from less well-supported organizations, from marginalized groups, and from regions of the world that experience tobacco-related inequities.

## Figures and Tables

**Table 1:** Characteristics of Training Program Trainees.

Number of Accredited Programs		Total	2019	2018	2017
		22	22	20	19
**Percent of Trainees Reporting Data**		86.8% (5203/5991)	97.9% (2227/2274)	90.5% (1656/1829)	69.9% (1320/1888)
**Variable**	**Range or Categories**				
Age (mean)	17–84	40.43 (12.33)	39.60 (12.49)	41.83 (12.01)	40.19 (12.29)
		N (%)	N (%)	N (%)	N (%)
Sex/Gender	Female	3785 (78.7)	1585 (77.8)	1181 (78.3)	1019 (80.6)
Race/Ethnicity	White/Caucasian	3063 (69.1)	1376 (70.8)	956 (68.7)	731 (66.5)
	Black/African American	604 (13.6)	259 (13.3)	200 (14.4)	145 (13.2)
	Asian	282 (6.4)	104 (5.4)	82 (5.9)	96 (8.7)
	American Indian/Alaska Native	95 (2.1)	30 (1.5)	29 (2.1)	36 (3.3)
	Native Hawaiian/Pacific Islander	31 (0.7)	16 (0.8)	14 (0.9)	1 (0.1)
	Hispanic of any race	358 (8.1)	158 (8.1)	110 (7.9)	90 (8.2)
Education level	High school or less	299 (7.0)	223 (10.7)	41 (2.8)	35 (4.9)
	Some college, associate degree, and other	535 (12.5)	257 (12.3)	198 (13.6)	77 (10.7)
	Bachelor’s degree	1398 (32.7)	681 (32.7)	491 (33.3)	226 (31.5)
	Master’s degree	1486 (34.8)	655 (31.5)	554 (37.6)	277 (38.6)
	Doctoral degree	554 (13.0)	265 (12.7)	186 (12.6)	103 (14.3)
Residence	North America	4519 (92.6)	2023 (91.8)	1513 (92.3)	983 (94.6)
	Middle East	297 (6.1)	160 (7.3)	87 (5.3)	50 (4.8)
	Europe	38 (0.8)	7 (0.3)	27 (1.6)	4 (0.4)
	Asia	15 (0.3)	7 (0.3)	6 (0.4)	2 (0.2)
	Africa	11 (0.2)	6 (0.3)	5 (0.3)	-
	Australia	1 (0.0)	-	1 (0.1)	-
Years working in tobacco treatment	0–1 years	2583 (66.2)	1437 (70.4)	791 (60.7)	355 (63.7)
	2–3 years	509 (13.0)	240 (11.8)	196 (15.0)	73 (13.1)
	4–5 years	268 (6.9)	129 (6.3)	92 (7.1)	47 (8.4)
	6–10 years	256 (6.6)	110 (5.4)	104 (8.0)	42 (7.5)
	11–15 years	128 (3.3)	59 (2.9)	45 (3.5)	24 (4.3)
	16 years or more	158 (4.0)	3.3 (67)	5.8 (75)	2.9 (16)
Hours per week working in tobacco treatment	None	1735 (41.4)	942 (47.2)	507 (39.9)	(30.8) 286
	1–7 h	1607 (38.3)	718 (36.0)	468 (36.8)	421 (45.4)
	8–16 h	267 (6.4)	104 (5.2)	95 (7.5)	68 (7.3)
	17–32 h	224 (5.3)	91 (4.6)	72 (5.7)	61 (6.6)
	>33 h	361 (8.6)	140 (7.0)	129 (10.1)	92 (9.9)
Reasons for seeking training *	To implement tobacco treatment services	2785 (61.0)	1326 (62.3)	874 (62.9)	585 (56.1)
	General interest/Learn	2537 (55.6)	1199 (56.3)	828 (59.6)	510 (48.9)
	Want to become certified	2378 (52.1)	1159 (54.4)	776 (55.8)	443 (42.5)
	Work related or work requirement	801 (17.6)	421 (19.8)	189 (13.6)	191 (18.3)
	Research and evaluation	721 (15.8)	314 (14.7)	292 (21.0)	115 (11.0)

* More than one reason can be selected.

**Table 2:** Number of Trainees by Training Program 2016–2019.

Program Name (Alpha Order)	Total (7761)	2019 (2274)	2018 (1829)	2017 (1888)	2016 (1770)
	N (%)	N (%)	N (%)	N (%)	N (%)
Alaska Native Tribal Health Consortium	128 (1.6)	29 (1.3)	26 (1.4)	33 (1.7)	40 (2.3)
Arizona Tobacco Treatment Specialist Course	33 (0.4)	0	33 (1.8)	*	*
BREATHE Online Tobacco Treatment Specialist Training Program	216 (2.8)	99 (4.4)	86 (4.7)	31 (1.6)	*
The Breathing Association	299 (3.9)	68 (3.0)	106 (5.8)	75 (4.0)	50 (2.8)
Duke-UNC Tobacco Treatment Specialist Training Program	365 (4.7)	120 (5.3)	153 (8.4)	92 (4.9)	*
Tobacco Treatment Specialist Training and Certificate Program, Duquesne University	430 (5.5)	230 (10.1)	77 (4.2)	50 (2.6)	73 (4.1)
Healthyways, a Sharecare Company	41 (0.5)	0	0	6 (0.3)	35 (2.0)
King Hussein Cancer Center Tobacco Dependence Treatment Training	192 (2.5)	118 (5.2)	74 (4.0)	*	*
Tobacco Treatment Education & Training Program, MaineHealth Center for Tobacco Independence	49 (0.6)	5 (0.2)	8 (0.4)	18 (1.0)	18 (1.0)
Tobacco Treatment Specialist Training, Mayo Clinic Nicotine Dependence Center	915 (11.8)	226 (9.9)	186 (10.2)	235 (12.4)	268 (15.1)
National Jewish Health^®^	93 (1.2)	33 (1.5)	38 (2.1)	14 (0.7)	8 (0.5)
Optum’s Quit For Life Program^®^	81 (1.0)	15 (0.7)	6 (0.3)	14 (0.7)	46 (2.6)
Rocky Mountain Tobacco Treatment Specialist Training Program	300 (3.9)	108 (4.7)	16 (0.9)	78 (4.1)	98 (5.5)
Roswell Park Tobacco Treatment Specialist Training Program	103 (1.3)	87 (3.8)	16 (0.9)	*	*
Rutgers Tobacco Dependence Program	425 (5.5)	149 (6.6)	93 (5.1)	81 (4.3)	102 (5.8)
Tobacco Treatment Specialist Course, Florida State University	560 (7.2)	87 (3.8)	104 (5.7)	120 (6.4)	249 (14.1)
Tobacco Treatment Specialist Training and Certification Program, College of Medicine, Taibah University	36 (0.5)	36 (1.6)	*	*	*
Tobacco Treatment Specialist Training Program, University of Massachusetts Medical School	2101 (27.1)	318 (14.0)	354 (19.4)	790 (41.8)	639 (36.1)
ACT Center for Tobacco Treatment, Education and Research, University of Mississippi Medical Center Cancer Institute	608 (7.8)	139 (6.1)	208 (11.4)	162 (8.6)	99 (5.6)
Comprehensive Smoking Treatment Program, University of Pennsylvania	180 (2.3)	51 (2.2)	48 (2.6)	36 (1.9)	45 (2.5)
Tobacco Treatment Training Program, The University of Texas MD Anderson Cancer	457 (5.9)	246 (10.8)	158 (8.6)	53 (2.8)	*
Tobacco Treatment Training Program, School of Dentistry, West Virginia University	149 (1.9)	110 (4.8)	39 (2.1)	*	*

* Not yet accredited that year.

**Table 3:** Tobacco Treatment Training Programs Accredited by the Council for Tobacco Treatment Training Programs through 2020.

Program Name	Year Accredited	Program Description
Tobacco Prevention and Control Program, Wellness and Prevention Department, Alaska Native Tribal Health Consortium (ANTHC), Anchorage, AK	2008	The ANTHC training program aids health care professionals develop and understand the required skills in a manner that reflects the Alaska Native spirit and addresses tobacco-related disparities in Alaska. Delivered in-person (annually) and virtually (quarterly), to date, ANTHC has trained over 500 professionals.
Arizona Tobacco Treatment Specialist Course, Mercy Care, Phoenix, AZ	2019	As a Medicaid health plan serving members in Maricopa County Arizona, Mercy Care’s training program focuses on providing high-quality evidence-based treatment for Tobacco Use Disorder (TUD) to plan members with serious mental illness. The Mercy Care training program’s mission is to train an internal treatment workforce to deliver high-quality evidence-based treatment for TUD to plan members.
BREATHE Online Tobacco Treatment Specialist Training Program, College of Nursing, University of Kentucky, Lexington, KY	2017	The BREATHE training program offers an asynchronous, fully online, self-paced training program in which participants receive written and virtual feedback on assignments using the Canvas learning platform. Developed to meet the needs of diverse professionals nationally and internationally, the training model and modality allows professionals to tailor a learning plan to their interests and settings.
Tobacco Treatment Specialist Certification Program, The Breathing Association, Columbus, OH	2016	The Breathing Association training program is focused on preparing health care and mental health professionals to provide treatment for TUD in diverse clinical and community settings. The curriculum also focuses on best practices in the development of tobacco treatment programs.
Duke-UNC Treatment Specialist Training Program, Durham, NC	2017	The Duke-UNC training program is a collaborative partnership between Duke University Smoking Cessation Program, UNC Tobacco Intervention Programs, and the Tobacco Prevention and Control Branch of the North Carolina Division of Public Health. The Duke-UNC training program offers highly interactive in-person and virtual training using a combination of didactic learning, interactive exercises, and case studies. The curriculum also provides training in tobacco control policy, and guidance on developing effective smoking cessation programs in diverse settings.
Tobacco Treatment Specialist Training and Certificate Program, Duquesne University, Pittsburg, PA	2014	Taught by faculty pharmacists, the Duquesne training program offers an enriched and unique perspective into the pharmacotherapy used for tobacco cessation.
Healthways, a Sharecare Company, Franklin, TN	2012	Healthways has delivered an internal wellness and tobacco cessation program since 2006. Healthways training program is focused on training health and wellness Coaches who work within the Healthways spectrum of services.
King Hussein Cancer Center (KHCC) Tobacco Dependence Treatment Training, Amman, Jordan	2017	The KHCC training program is focused on building the capacity, competence, and confidence of professionals in Jordan and other countries in the Eastern Mediterranean region. The KHCC training is available in-person and virtually. The virtual training aims to build a wide network of tobacco treatment providers across the region, provide a robust exchange of experiences, and sustain the tobacco dependence treatment efforts in Jordan and the Eastern Mediterranean region.
Tobacco Treatment Education & Training Program, MaineHealth Center for Tobacco Independence, Portland, ME	2012	The MaineHealth training program is an integral part of a coordinated, collaborative statewide effort to address tobacco use and exposure through education, prevention, policy, treatment, and training. The MaineHealth mission is “Making our communities the healthiest in America through reduction of tobacco use and through the provision of evidence-based treatment, education, policy development and research.” The MaineHealth training focuses on developing a workforce of highly trained Tobacco Treatment Specialists in Maine.
Tobacco Treatment Specialist Training, Mayo Clinic Nicotine Dependence Center, Program, Rochester, MN	2010	Since 2010, the Mayo Clinic training program has trained over 3000 professionals. The Mayo Clinic training program offers a unique blended learning experience which includes synchronous and asynchronous virtual learning and/or in-person training modalities. The Mayo Clinic program is focused on preparing professionals to treat tobacco dependence in diverse settings including hospitals, community health centers, dental practices, public health organizations, telephone quit lines, and addiction and mental health centers.
National Jewish Health^®^, Denver, CO	2015	National Jewish Health^®^ is the leading respiratory hospital in the United States and the largest nonprofit provider of telephone quitline services. National Jewish Health’s training program is focused on training professionals within the National Jewish Health^®^ organization including all the Health and Wellness Coaches and the quitline Quit Coaches, and other employees. Professionals are trained to deliver in-person, telephone, text, and chat-based interventions to quitline participants and patients in the National Jewish Health^®^ hospitals and clinics.
Optum’s Quit For Life Program^®^, Optum, Eden Prairie, MN	2016	Optum is a leading health services innovation company. The Quit For Life^®^ training program is offered to Quit Coaches who support Optum’s Quit For Life Quit^®^ treatment services. The Quit For Life program has over 30 years of experience operating tobacco quitline services to health plans, employers and state quitlines. Optum’s virtual training uses synchronous and asynchronous methods to prepare Quit Coaches to deliver interventions via telephone, text, chat and online groups to a diverse population including adults, youth, and vulnerable populations such as those with behavioral health conditions.
Rocky Mountain Tobacco Treatment Specialist (RMTTS) Training Program, Department of Psychiatry, University of Colorado Anschutz Medical Campus, Aurora, CO	2015	The RMTTS training program is part of the Behavioral Health and Wellness Program at the University of Colorado, a multi-disciplinary center of excellence for public policy, research, training, and clinical care. The RMTTS training program’s mission is to train interdisciplinary health care providers and community and public health professionals to become tobacco cessation champions in their organizations and communities. The RMTTS training program works with communities, healthcare facilities, and public health and governmental agencies to promote tobacco treatment as part of overall health and wellness in priority populations.
Roswell Park Tobacco Treatment Specialist Training Program, Roswell Park Comprehensive Cancer Center, Buffalo, NY	2018	The mission of the Roswell Park training program is to contribute to the professional development of the tobacco treatment clinical and research workforce in Western New York, in the United States, and across the world. The Roswell Park program is also focused on training Roswell Park Cessation Services Quit Coaches. The curriculum focuses on individual, telephone, and virtual treatment modalities, provides hands-on experience delivering an evidence-based treatment manual, and is enhanced with in-depth content about tobacco product marketing, tobacco product development, and tobacco regulatory science.
Rutgers Tobacco Dependence Program, Center for Tobacco Studies, Cancer Institute of New Jersey, Robert Wood Johnson Medical School, School of Public Health, New Brunswick, NJ	2010	The Rutgers training program has been training tobacco treatment specialists since 2001, with a multidisciplinary training faculty consisting of physicians, behavioral health clinicians, and public health experts. The Rutgers program is dedicated to reducing the harm caused by tobacco use for those who need it most, by training professionals to provide education and evidence-based treatment, conduct innovative research, and engage in advocacy.
Tobacco Treatment Specialist Course, College of Medicine, Florida State University (FSU), Tallahassee, FL	2011	Since accreditation, the FSU program has trained over 1100 professionals and paraprofessionals. Training is provided by an interdisciplinary faculty of physicians, clinicians, and public health experts with extensive experience in treating tobacco use disorders and tobacco control. The goal of the FSU program is to prepare TTSs to treat tobacco users, provide training to others, act as consultants in addressing challenging situations, and implement systems change strategies in health systems and behavioral health programs.
Tobacco Treatment Specialist Training and Certification Program (TTS-TCP), College of Medicine, Taibah University, Madinah Saudi Arabia	2019	The TTS-TCP aims to provide comprehensive, practical and standardized training in tobacco treatment and research for health care professionals in Saudi Arabia, the Gulf Cooperation Council (GCC) countries, and the wider region. Taibah University aims to support the GCC efforts to address a continuous increase in the prevalence of tobacco smoking in the Gulf region by providing high-quality, standardized training in tobacco treatment. Although the primary focus of the Taibah program is to train health care workers in Saudi Arabia, it aims to be a resource for workers in the neighboring countries. In fact, professionals from other GCC countries were among the attendees of the first course in Taibah University in October of 2019.
Tobacco Treatment Specialist Training Program, Center for Tobacco Treatment Research and Training, University of Massachusetts Medical School, Worcester, MA	2012	Founded in 1999, the UMass training program offers blended synchronous and asynchronous virtual and in-person training modalities. The mission of the UMass program is to increase access to evidence-based tobacco dependence treatment services across the world. The UMass program has a unique structure with workshops delivered by UMass faculty and UMass Certified Trainers located in diverse settings throughout the world.
ACT Center for Tobacco Treatment, Education and Research, University of Mississippi Medical Center Cancer Institute, Jackson, MS	2011	The ACT Center has trained TTSs in Mississippi across the US and internationally since 1999. This program attracts a broad range of professionals who work in a variety of health care, academic, community, and other settings. A unique aspect of this program is that clinical skills are taught within the context of hands-on mastery of the ACT Center Tobacco Treatment clinical treatment protocol. Substantial time is dedicated to hands-on practice delivering this treatment protocol. This manualized, evidence-based approach is the product of many years of testing and experience and represents an effective balance of state-of-the-art clinical procedures.
Comprehensive Smoking Treatment Program, Perelman School of Medicine, University of Pennsylvania, Philadelphia, PA	2013	The University of Pennsylvania’s training program is highly interactive and delivered in both in-person and virtual training modalities. The University of Pennsylvania program is focused on cultivating a deep understanding of the biological, social, and environmental factors that influence the tobacco epidemic. The program expects trainees to develop new perspectives on tobacco dependence and experience fundamental changes in their approaches to the treatment of tobacco use and dependence.
Tobacco Treatment Training Program, The University of Texas MD Anderson Cancer Center, Houston, TX	2017	The MD Anderson training program is focused on training multidisciplinary health care providers as well as community and public health professionals seeking to become tobacco treatment and nicotine addiction specialists and champions for their organizations and communities. The MD Anderson program offers onsite, traveling team, and remotely delivered training. The program also offers unique opportunities for on-going support and continuous education for all TTSs through weekly ECHO tele-mentoring sessions, called Project TEACH.
Tobacco Treatment Training Program, School of Dentistry, West Virginia University, Morgantown, WV	2017	The WVU training program teaches evidence-based tobacco treatment to a wide variety of healthcare professionals. This mission is accomplished with a multidisciplinary team of expert faculty from dentistry, medicine, nursing, pharmacy, social work, and public health. Training is provided in a three-day in-person workshop but also will be provided as part of the required curricula of the WVU Schools of Dentistry, Medicine, Nursing, Pharmacy, and Public Health.
Tobacco Treatment Specialist Training Program, Memorial Sloan Kettering Cancer Center, New York, NY	2020	The MSK training program is focused on training clinicians and clinical researchers. Utilizing the MSK Comskills Training Laboratory, the MSK training program utilizes a unique blend of hands-on learning with professional actors who simulate live patient scenarios. Training is delivered in synchronous virtual and in-person training modalities.

**Table 4:** Primary professional background of trainees (*n* = 3848).

Profession	Number (Percent)
Nursing	671 (17.4)
Social Work	361 (9.4)
Health Education	310 (8.1)
Physician	275 (7.1)
Mental Health Counselor or Specialist—non-doctoral	260 (6.8)
Advanced Practice Nurse or Physician Assistant	255 (6.6)
Respiratory Therapy	252 (6.5)
Pharmacy Professional	215 (5.6)
Psychologist	204 (5.3)
Alcohol and Substance Use Treatment Professional	163 (4.2)
Public Health Professional	120 (3.1)
Administration or Management Professional	47 (1.2)
Dental Professional	46 (1.2)
Other *	669 (17.4)

* Reported by less than 1% of respondents, but includes such diverse professionals as health and wellness coaches, registered dieticians, traditional or complementary medical professionals, and radiologic technologists.

**Table 5:** Trainee primary work setting (*n* = 4629).

Work Setting	Number (Percent)
Hospital, Medical, or Academic Medical Center	2335 (50.4)
Community Health Center	528 (11.4)
Public Health	408 (8.8)
Counseling Centers	316 (6.8)
Tobacco Cessation Treatment Programs	268 (5.8)
Addiction Treatment	242 (5.2)
Wellness Program	171 (3.7)
Social Service Agency	43 (1.0)
Other *	318 (6.9)

* Reported by less than 1% of respondents, but includes such diverse work settings as dental practices, government agencies, homeless shelters, research, health insurance companies, and the military.

## Data Availability

The data presented in this study are available on request and agreement with the Council for Tobacco Treatment Training Programs, Inc. The data are not publicly available but accessible with the appropriate data use agreement with the Council. Data requests should be made to info@ctttp.org.

## Supplementary Materials

The following are available online at https://www.mdpi.com/1660-4601/18/5/2416/s1, Document S1: Tobacco Product Use Common in Certain Regions of the World. References [67,68,69,70,71,72,73,74] are cited in the supplementary materials.

## References

[1] AAAP (2015). Nicotine Dependence in Translating Science. Transforming Lives.

[2] Gowing L.R., Ali R.L., Allsop S., Marsden J., Turf E.E., West R., Witton J. (2015). Global statistics on addictive behaviours: 2014 status report. Addiction.

[3] WHO (2017). WHO Report on the Global Tobacco Epidemic, 2017: Monitoring Tobacco Use and Prevention Policies.

[4] Öberg M. (2010). Second-hand smoke: Assessing the environmental burden of disease at national and local levels. WHO Environmental Burden of Disease Series.

[5] Öberg M., Jaakkola M.S., Woodward A., Peruga A., Prüss-Ustün A. (2011). Worldwide burden of disease from exposure to second-hand smoke: A retrospective analysis of data from 192 countries. Lancet.

[6] WHO Tobacco. https://www.who.int/news-room/fact-sheets/detail/tobacco.

[7] Jha P. (2009). Avoidable global cancer deaths and total deaths from smoking. Nat. Rev. Cancer.

[8] Mathers C.D., Loncar D. (2006). Projections of Global Mortality and Burden of Disease from 2002 to 2030. PLoS Med..

[9] Goodchild M., Nargis N., D’Espaignet E.T. (2018). Global economic cost of smoking-attributable diseases. Tob. Control..

[10] Goodchild M.F., Guo H., Annoni A., Bian L., De Bie K., Campbell F., Craglia M., Ehlers M., Van Genderen J., Jackson D. (2012). Next-generation Digital Earth. Proc. Natl. Acad. Sci. USA.

[11] WHO Regional Office for the Eastern Mediterranean (2019). Jordan: Making the Economic Case for Tobacco Control Action in Jordan.

[12] Loring B. (2014). Tobacco and Inequities: Guidance for Addressing Inequities in Tobacco-Related Harm.

[13] Barnoya J., Glynn T. (2012). Reducing global health inequities through tobacco control. Cancer Causes Control..

[14] WHO (2003). WHO Framework Convention on Tobacco Control.

[15] Kruse G.R., Rigotti N.A., Raw M., McNeill A., Murray R., Piné-Abata H., Bitton A., McEwen A. (2015). Tobacco Dependence Treatment Training Programs: An International Survey. Nicotine Tob. Res..

[16] Khattab A., Javaid A., Iraqi G., Alzaabi A., Ben Kheder A., Koniski M.-L., Shahrour N., Taright S., Idrees M., Polatli M. (2012). Smoking habits in the Middle East and North Africa: Results of the BREATHE study. Respir. Med..

[17] WHO (2015). Advisory Note: Waterpipe Tobacco Smoking: Health Effects, Research Needs and Recommended Actions for Regulators.

[18] Al-Houqani M., Leinberger-Jabari A., Al Naeemi A., Al Junaibi A., Al Zaabi E., Oumeziane N., Kazim M., Al Maskari F., Al Dhaheri A., Wareth L.A. (2018). Patterns of tobacco use in the United Arab Emirates Healthy Future (UAEHFS) pilot study. PLoS ONE.

[19] WHO (2018). WHO Global Report on Trends in Prevalence of Tobacco Smoking 2000–2025.

[20] Hassounah S., Rawaf D., Khoja T., Rawaf S., Hussein M., Qidwai W., Majeed A. (2014). Tobacco control efforts in the Gulf Cooperation Council countries: Achievements and challenges. East. Mediterr. Health J..

[21] WHO Regional Office for the Eastern Mediterranean (2012). Regional Framework for Action to Implement the United Nations Political Declaration on Noncommunicable Diseases: Annex to Resolution EM/RC59/R.2.

[22] U.S. Department of Health and Human Services (1998). Tobacco Use Among U.S. Racial/Ethnic Minority Groups—African Americans, American Indians and Alaska Natives, Asian Americans and Pacific Islanders, and Hispanics: A Report of the Surgeon General.

[23] National Center for Chronic Disease Prevention and Health Promotion. https://www.cdc.gov/chronicdisease/index.htm.

[24] American Psychiatric Association (2014). Diagnostic and Statistical Manual of Mental Disorders.

[25] DHHS (2014). The Health Consequences of Smoking—50 Years of Progress. A Report of the Surgeon General.

[26] U.S. (2020). Department of Health and Human Services. Smoking Cessation. A Report of the Surgeon General. U.S. Department of Health and Human Services.

[27] Babb S. (2017). Quitting Smoking among Adults—United States, 2000–2015. MMWR Morb. Mortal Wkly. Rep..

[28] Abughosh S., Wu I.-H., Hawari F., Peters R.J., Yang M., Crutchley R., Essien E.J. (2011). Predictors of Intention to Quit Cigarette Smoking among Jordanian Adult. Epidemiol. Open Access.

[29] Zhu S.-H., Sun J.Y., Bonnevie E., Cummins S.E., Gamst A., Yin L., Lee M. (2014). Four hundred and sixty brands of e-cigarettes and counting: Implications for product regulation. Tob. Control..

[30] Bhatnagar A., Whitsel L.P., Blaha M.J., Huffman M.D., Krishan-Sarin S., Maa J., Rigotti N., Robertson R.M., Warner J.J., on behalf of the American Heart Association (2019). New and Emerging Tobacco Products and the Nicotine Endgame: The Role of Robust Regulation and Comprehensive Tobacco Control and Prevention: A Presidential Advisory from the American Heart Association. Circ..

[31] Anthonisen N.R. (2005). The effects of a smoking cessation intervention on 14.5-year mortality: A randomized clinical trial. Ann. Intern. Med..

[32] Fiore M.C. (2008). Treating tobacco use and dependence: 2008 update. Clinical Practice Guideline.

[33] Kasza K.A., Hyland A.J., Borland R., McNeill A.D., Bansal-Travers M., Fix B.V., Hammond D., Fong G.T., Cummings K.M. (2013). Effectiveness of stop-smoking medications: Findings from the International Tobacco Control (ITC) Four Country Survey. Addiction.

[34] Brose L.S., McEwen A., Michie S., West R., Chew X.Y., Lorencatto F. (2015). Treatment manuals, training and successful provision of stop smoking behavioural support. Behav. Res. Ther..

[35] Brose L.S., McEwen A., West R. (2012). Does it matter who you see to help you stop smoking? Short-term quit rates across specialist stop smoking practitioners in England. Addiction.

[36] Brose L.S., West R., McEwen A. (2014). How stable are stop smoking practitioner success rates over time?. Transl. Behav. Med..

[37] Brose L.S., West R., Michie S., McEwen A. (2014). Changes in success rates of smoking cessation treatment associated with take up of a national evidencebased training programme. Prev. Med..

[38] McDermott M.S., Beard E., Brose L.S., West R., McEwen A. (2012). Factors Associated With Differences in Quit Rates Between “Specialist” and “Community” Stop-Smoking Practitioners in the English Stop-Smoking Services. Nicotine Tob. Res..

[39] Brose L.S., West R., McDermott M.S., Fidler J.A., Croghan E., McEwen A. (2011). What makes for an effective stop-smoking service?. Thorax.

[40] WHO (2019). WHO Report on the Global Tobacco Epidemic.

[41] Warren G.W., Marshall J.R., Cummings K.M., Toll B., Gritz E.R., Hutson A., Dibaj S., Herbst R., Dresler C. (2013). Practice Patterns and Perceptions of Thoracic Oncology Providers on Tobacco Use and Cessation in Cancer Patients. J. Thorac. Oncol..

[42] Twardella D., Brenner H. (2005). Lack of training as a central barrier to the promotion of smoking cessation: A survey among general practitioners in Germany. Eur. J. Public Health.

[43] Jradi H. (2017). Awareness, practices, and barriers regarding smoking cessation treatment among physicians in Saudi Arabia. J. Addict. Dis..

[44] Rojewski A.M., Bailey S.R., Bernstein S.L., A Cooperman N., Gritz E.R., A Karam-Hage M., E Piper M., A Rigotti N., Warren G.W. (2019). Considering Systemic Barriers to Treating Tobacco Use in Clinical Settings in the United States. Nicotine Tob. Res..

[45] Kruse G.R., Rigotti N.A., Raw M., McNeill A., Murray R., Piné-Abata H., Bitton A., McEwen A. (2017). Content and Methods used to Train Tobacco Cessation Treatment Providers: An International Survey. J. Smok. Cessat..

[46] McDermott M.S., West R., Brose L.S., McEwen A. (2012). Self-reported practices, attitudes and levels of training of practitioners in the English NHS Stop Smoking Services. Addict. Behav..

[47] Stead M., Angus K., Holme I., Cohen D., Tait G. (2009). Factors influencing European GPs’ engagement in smoking cessation: A multi-country literature review. Br. J. Gen. Pr..

[48] Vogt F., Hall S., Marteau T.M. (2005). General practitioners’ and family physicians’ negative beliefs and attitudes towards discussing smoking cessation with patients: A systematic review. Addiction.

[49] Vogt F., Hall S., Marteau T.M. (2006). General practitioners’ beliefs about effectiveness and intentions to prescribe smoking cessation medications: Qualitative and quantitative studies. BMC Public Health.

[50] Kotz D., Brown J., West R. (2014). Prospective cohort study of the effectiveness of smoking cessation treatments used in the “real world”. Mayo Clin. Proc..

[51] Song F., Maskrey V., Blyth A., Brown T.J., Barton G.R., Aveyard P., Notley C., Holland R., Bachmann M.O., Sutton S. (2015). Differences in Longer-Term Smoking Abstinence After Treatment by Specialist or Nonspecialist Advisors: Secondary Analysis of Data From a Relapse Prevention Trial. Nicotine Tob. Res..

[52] Carter G.T., Javaher S.P., Nguyen M.H., Garret S., Carlini B.H. (2015). Re-branding cannabis: The next generation of chronic pain medicine?. Pain Manag..

[53] Kalkhoran S., Thorndike A.N., Rigotti N.A., Fung V., Baggett T.P. (2019). Cigarette Smoking and Quitting-Related Factors Among US Adult Health Center Patients with Serious Mental Illness. J. Gen. Intern. Med..

[54] Diamanti A., Papadakis S., Schoretsaniti S., Rovina N., Vivilaki V., Gratziou C., Katsaounou P.A. (2019). Smoking cessation in pregnancy: An update for maternity care practitioners. Tob. Induc. Dis..

[55] CAN-ADAPTT (2011). Canadian Smoking Cessation Clinical Practice Guideline.

[56] NICE (2013). Smoking Cessation: Supporting People to Stop Smoking.

[57] Health M.O. (2017). New Zealand Smoking Cessation Guidelines.

[58] Schauer G.L. (2016). Health-care Provider Screening and Advice for Smoking Cessation among Smokers with and without COPD: 2009-2010 National Adult Tobacco Survey. Chest.

[59] Vidrine J.I. (2013). Ask-Advise-Connect: A new approach to smoking treatment delivery in health care settings. JAMA Intern. Med..

[60] Vidrine J.I. (2013). The Ask-Advise-Connect approach for smokers in a safety net healthcare system: A group-randomized trial. Am. J. Prev. Med..

[61] Sheffer C.E., Stein J.S., Petrucci C., Mahoney M.C., Johnson S., Giesie P., Carl E., Krupski L., Tegge A.N., Reid M.E. (2020). Tobacco Dependence Treatment in Oncology: Initial Patient Clinical Characteristics and Outcomes from Roswell Park Comprehensive Cancer Center. Int. J. Environ. Res. Public Health.

[62] Burke M.V., Ebbert J.O., Schroeder D.R., McFadden D.D., Hays J.T. (2015). Treatment Outcomes from a Specialist Model for Treating Tobacco Use Disorder in a Medical Center. Medicine (Baltim.).

[63] Joseph A.M., Rothman A.J., Almirall D., Begnaud A., Chiles C., Cinciripini P.M., Fu S.S., Graham A.L., Lindgren B.R., Melzer A.C. (2018). Lung Cancer Screening and Smoking Cessation Clinical Trials. SCALE (Smoking Cessation within the Context of Lung Cancer Screening) Collaboration. Am. J. Respir. Crit. Care Med..

[64] Bitton A., Raw M., Richards A., McNeill A., Rigotti N.A. (2010). A comparison of four international surveys of tobacco dependence treatment provision: Implications for monitoring the Framework Convention on Tobacco Control. Addiction..

[65] Sheffer C.E. (2015). Increasing the quality and availability of evidence-based treatment for tobacco dependence through unified cerification of tobacco treatment specialists. J. Smok. Cessat..

[66] Fagan P., Moolchan E.T., Lawrence D., Fernander A., Ponder P.K. (2007). Identifying health disparities across the tobacco continuum. Addiction.

[67] Kyaing N., Sinha D., Islam M., Rinchen S. (2011). Social, economic and legal dimensions of tobacco and its control in South-East Asia region. Indian J. Public Health.

[68] Sreeramareddy C.T., Pradhan P.M.S., Mir I.A., Sin S. (2014). Smoking and smokeless tobacco use in nine South and Southeast Asian countries: Prevalence estimates and social determinants from Demographic and Health Surveys. Popul. Health Metrics.

[69] Vupputuri S., Hajat C., Al-Houqani M., Osman O., Sreedharan J., Ali R., E Crookes A., Zhou S., E Sherman S., Weitzman M. (2014). Midwakh/dokha tobacco use in the Middle East: Much to learn. Tob. Control..

[70] Tabuchi T., Gallus S., Shinozaki T., Nakaya T., Kunugita N., Colwell B. (2018). Heat-not-burn tobacco product use in Japan: Its prevalence, predictors and perceived symptoms from exposure to secondhand heat-not-burn tobacco aerosol. Tob. Control..

[71] Ali F.R.M., Diaz M.C., Vallone D., Tynan M.A., Cordova J., Seaman E.L., Trivers K.F., Schillo B.A., Talley B., King B.A. (2020). E-cigarette Unit Sales, by Product and Flavor Type—United States, 2014–2020. MMWR Morb. Mortal. Wkly. Rep..

[72] Weaver S.R., Majeed B.A., Pechacek T.F., Nyman A.L., Gregory K.R., Eriksen M.P. (2016). Use of electronic nicotine delivery systems and other tobacco products among USA adults, 2014: Results from a national survey. Int. J. Public Health.

[73] Llp A.A. Electronic Cigarette Market by Product Type, Flavor and Distribution Channel—Global Opportunity Analysis and Industry Forecast 2017–2023. https://www.researchandmarkets.com/reports/4458933/electronic-cigarette-market-by-product-type.

[74] Grana R.A., Ling P.M. (2014). Smoking Revolution: A Content Analysis of Electronic Cigarette Retail Websites. Am. J. Prev. Med..

