# Supplementary material for: The Emerging Global Tobacco Treatment Workforce: Characteristics of Tobacco Treatment Specialists Trained in Council-Accredited Training Programs from 2017 to 2019
Source: Int J Environ Res Public Health. 2021 Mar 2;18(5):2416. doi: 10.3390/ijerph18052416 (PMC7967787; doi:10.3390/ijerph18052416)
Supplement: Supplementary file 1 [file ijerph-18-02416-s001.pdf]

Supplement Document S1: Tobacco Product Use Common in Certain Regions of the World

The following are examples of some of the diverse tobacco products common in different regions of the world and worldwide. In Southeast Asia, popular tobacco products include chewing khaini (tobacco with slaked lime and aromatic spices), surti (dried tobacco leaves for chewing), or paan masala (tobacco with aromatic spices), sucking gutkha (mixture of tobacco and molasses available in small sachets), applying gul or gudaku as dentifrice, and inhaling nas and naswar (nasal inhalation of tobacco powder). [67,68] In the Middle East, popular tobacco use options include hookah or water pipes, eastern bidis (thin cigarettes wrapped in the tendu or temburni leaf), kreteks (clove cigarettes), pan (betel quid with tobacco), and midwakh or dokha smoked in a pipe (tobacco blended with a variety of barks, herbs, spices, dried flowers or dried fruit) [69]. In Japan, the use of IQOS, a heat-not-burn tobacco product, increased 10-fold from 2015–2017. [70] Each product has different levels and rapidity of nicotine delivery, different use patterns and associated behaviors, and different cultural influences. Many individuals use more than one product making effective assessment and treatment of TUD increasingly complex.

The global proliferation of flavored ENDS is of particular concern. ENDS are battery-powered devices designed to deliver doses of nicotine and other additives via an aerosol. Total ENDS sales increased by 300% from 2016 to 2019. [71] Unfortunately, the use of ENDS appears to be highest among young adults and adults with poorer health and multiple comorbid conditions, [72] and despite a lack evidence for safety and efficacy for quitting, many cigarette smokers begin using ENDS as a way to quit smoking. [73] The increase in ENDS use is partly due to misleading marketing on behalf of the global tobacco industry. An analysis of websites aimed at selling ENDS products found that 95% made unsubstantiated health-related claims, 64% claimed their products were helpful for smoking cessation, 22% featured a physician's recommendation, and 76% claimed the products did not produce secondhand exposure. [74] These websites frequently mention that products are "cleaner" and "cheaper" than cigarettes, can be smoked anywhere, and can be used as a "workaround" to clean air policies. [74] Detailed knowledge of this changing landscape and the accuracy of the claims made by advertising for these products is required to engage in meaningful and effective discussions with tobacco users, particularly those seeking to quit tobacco.
